# Single-cell RNA sequencing confirms IgG transcription and limited diversity of V_H_DJ_H_ rearrangements in proximal tubular epithelial cells

**DOI:** 10.1038/s41598-020-75013-9

**Published:** 2020-11-12

**Authors:** Zhenling Deng, Xinyao Wang, Yue Liu, Xinyu Tian, Shaohui Deng, Yingchun Sun, Song Wang, Danxia Zheng, Zhuan Cui, Yuejuan Pan, Lata A, Huige Yan, Xiaoyan Qiu, Yue Wang

**Affiliations:** 1 Department of Nephrology, Peking University Third Hospital, 49 Huayuanbei Road, Beijing, 100191 People’s Republic of China; 2 Department of Immunology, School of Basic Medical Sciences, Peking University, 38 Xueyuan Road, Beijing, 100191 People’s Republic of China

**Keywords:** Immunology, Nephrology

## Abstract

Increasing evidence has confirmed that immunoglobulins (Igs) can be expressed in non-B cells. Our previous work demonstrated that mesangial cells and podocytes express IgA and IgG, respectively. The aim of this work was to reveal whether proximal tubular epithelial cells (PTECs) express Igs. High-throughput single-cell RNA sequencing (scRNA-seq) detected Igs in a small number of PTECs, and then we combined nested PCR with Sanger sequencing to detect the transcripts and characterize the repertoires of Igs in PTECs. We sorted PTECs from the normal renal cortex of two patients with renal cancer by FACS and further confirmed their identify by *LRP2* gene expression. Only the transcripts of the IgG heavy chain were successfully amplified in 91/111 single PTECs. We cloned and sequenced 469 V_H_DJ_H_ transcripts from 91 single PTECs and found that PTEC-derived IgG exhibited classic V_H_DJ_H_ rearrangements with nucleotide additions at the junctions and somatic hypermutations. Compared with B cell-derived IgG, PTEC-derived IgG displayed less diversity of V_H_DJ_H_ rearrangements, predominant VH1-24/DH2-15/JH4 sequences, biased VH1 usage, centralized VH gene segment location at the 3′ end of the genome and non-Gaussian distribution of the CDR3 length. These results demonstrate that PTECs can express a distinct IgG repertoire that may have implications for their role in the renal tubular epithelial-mesenchymal transition.

## Introduction

Proximal tubular epithelial cells (PTECs) are the most abundant cell type in the kidney and have the functions of reabsorption, concentration, exocrine as well as endocrine and metabolism under normal conditions^1^. Kidney tubular epithelial cells express multiple Toll-like receptors (TLR1, 2, 3, 4, 9)^2^. Upon exposure to pathogens, PTECs produce growth factors and a repertoire of inflammatory molecules, including cytokines (IL-1β, IL-6, IL-18 and TNF-α), chemokines (CCL2, CCL3 and CCL5), and complement components (C2, C3, C4 and C1q); further, PTECs play an important role in regaining tissue integrity or promoting progression to chronic kidney diseases^3^. However, to date, the expression of immunoglobulins (Igs) by PTECs has not been reported.

Traditionally, the expression of Igs has been thought to be exclusive to B cells. However, this theory has been challenged over the past decade by increasing evidence reporting that Igs could be expressed in non-B cells. Initially, it was found that human cancers, including breast, colon, lung, prostate, esophagus, kidney, bladder, and some cancer cell lines, can produce Igs in both cytoplasmic and secretory forms^4–10^. Subsequently, much evidence has revealed that Igs can also be synthesized by normal non-B cells, even these so-called immune privileged sites, including the eyes, central neurons, placenta, and testes, as well as mammary epithelial cells during lactation^11–16^. More importantly, in B cell-deficient μMT mice, different classes of Igs were detected in cardiomyocytes^17^ and liver epithelial cells^18^.

In contrast to B-Igs, non-B-Igs displayed unique V(D)J recombination patterns and physicochemical properties, such as abnormal glycation^7^ and hydrophobic properties. Functionally, non-B-Igs can serve not only as natural antibodies in skin and mucosa^19,20^ but also as growth factors to promote cell proliferation and adhesion as well as promote initiation and metastasis of cancer by binding to integrins as extracellular matrix protein^7^.

Our previous studies showed that cultured human mesangial cells (HMCs, both primary cells and cell lines)^21^ and human podocyte cell line (HPC)^22^ can synthesize and secrete IgA and IgG, respectively. HMC- and HPC-derived Igs exhibited conservative V(D)J patterns in the variable regions, which were consistent with the patterns of non-B-Igs, and they played an important role in cell proliferation and adhesion.

To obtain direct evidence of Ig gene transcription and rearrangements in PTECs, a 10 × Genomics Chromium Single Cell Gene Expression was first utilized, and IgG gene transcripts without V(D)J rearrangements were detected in only a few PTECs. We then turned to nested PCR combined with Sanger sequencing and successfully amplified IgG gene transcripts in abundant PTECs. Analysis of V_H_DJ_H_ rearrangements showed that PTEC-derived IgG displayed not only classic V_H_DJ_H_ rearrangements with somatic hypermutations but also biased VH1 usage and predominant VH1-24/DH2-15/JH4 sequences.

## Results

### IgG transcripts were detected in only a small number of PTECs by high-throughput scRNA-seq

**Figure 1:**
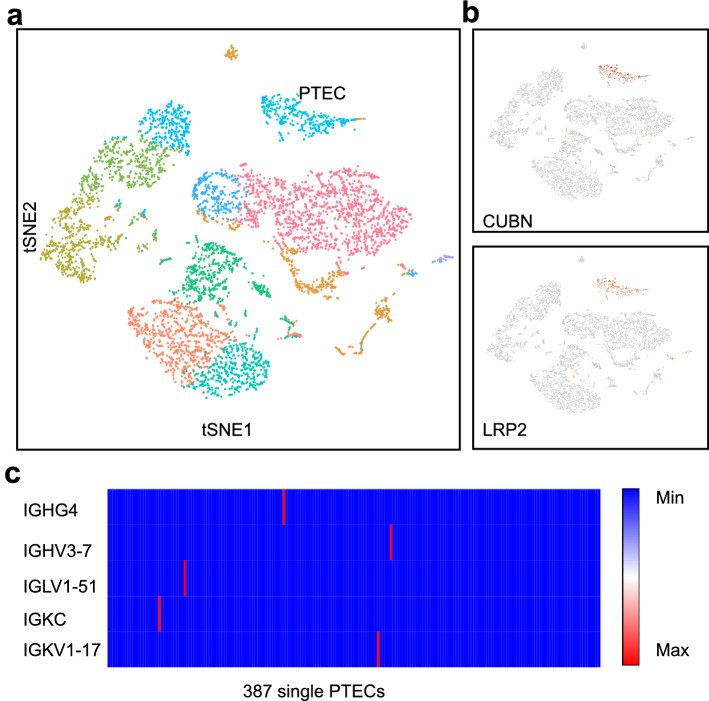
Few Ig transcripts were detected in PTECs by high-throughput single-cell sequencing. (**a**) The plot shows a two-dimensional representation (*tSNE* t-distributed stochastic neighbor embedding) of global relationships among 6138 single cells. A total of 11 clusters were identified, among which PTECs accounted for 6.1% (387/6138). Loupe Browser (version 4.0.0) was used for clustering. (**b**) Expression of PTEC marker genes (CUBN and LRP2) colored on the basis of normalized expression levels (gray, low; red, high). (**c**) Only five Ig transcripts were detected in five PTECs. The horizontal axis represents 387 single PTECs and the vertical axis represents five Ig genes (blue, no expression; red, expression).

We first prepared a single-cell suspension by enzymatic digestion of the normal renal cortex from a patient undergoing nephrectomy as a result of renal carcinoma; then, we performed scRNA-seq and V(D)J-seq using a Single-Cell Immune Profiling Solution. As shown in Fig. 1, unsupervised clustering analysis of the single cells using Loupe Browser identified 11 distinct cell clusters. On the basis of the specific marker genes LRP2 and CUBN, 387 single cells were defined as PTECs. IgG genes (both heavy chain and light chain as well as the constant region and variable region), IGHG4, IGHV3-7, IGKC, IGKV1-17 and IGLV1-51, were detected in only five single PTECs without V(D)J rearrangements detected.

### IgG transcripts were amplified by nested PCR of single isolated PTECs

**Figure 2:**
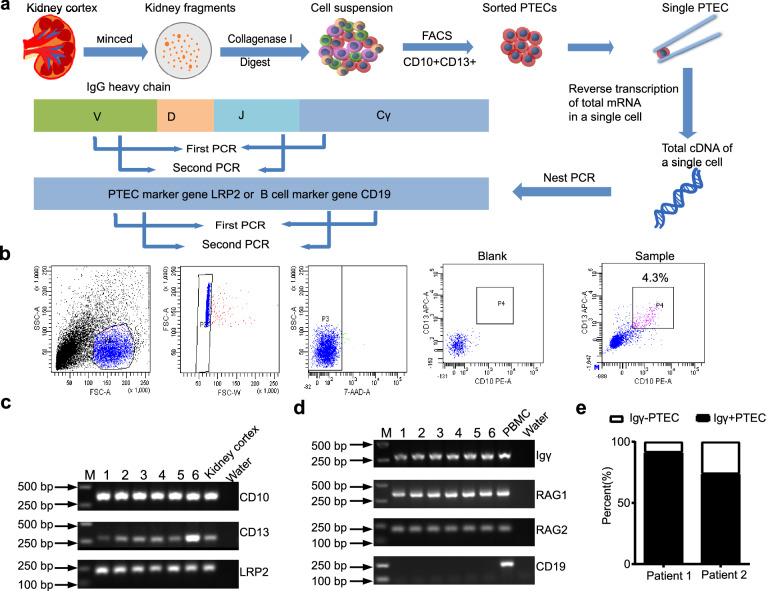
Transcripts of the Igγ chain were detected in sorted PTECs by FACS. (**a**) Schematic overview of the single-cell sequencing workflow in PTECs. (**b**) PTECs were sorted by FACS using antibodies against CD10-PE and CD13-APC. FACS analysis revealed that 4.3% of cells were double-positive. (**c**) PCR analysis of three PTEC marker genes: CD10, CD13 and LRP2; cDNA from the kidney cortex was used as a positive control. (**d**) PCR analysis of Igγ, RAG1, RAG2 and the B cell marker gene CD19. Peripheral blood mononuclear cells (PBMCs) were used as the positive control. Water instead of cDNA was used as a negative control. The Numbers (1 to 6) refer to six randomly selected PTEC cells for presentation. The full-length gels were presented in Supplementary Fig. 1. (**e**) IgG positive detection rate in PTECs from 2 patients was 91.8%, 74.2%, respectively.

Given that it was difficult to detect low-abundance genes by high-throughput scRNA-seq, we turned to nested PCR combined with Sanger sequencing to detect the transcription and V(D)J rearrangement of Igs in single PTECs. The renal cortex was collected from fresh nephrectomy specimens collected from renal carcinoma patients (n = 2). The normal renal cortex was removed far from the tumor to ensure the absence of cancer and significant parenchymal lesions (Table S1). PTECs were isolated from the human renal cortex through co-labeling of CD10 and CD13, two renal proximal tubular epithelial markers, and sorting by flow cytometry^23^. As shown in Fig. 2, 111 CD10/CD13 double-positive PTECs were manually picked under the microscope and were confirmed to be PTECs because of their expression of the PTEC-specific marker gene LRP2 (Fig. 2c and Fig. S1); contaminating B cells were excluded based on CD19 expression (Fig. 2d and Fig. S1), and the resultant cells were analyzed for Ig transcription and V_H_DJ_H_ rearrangements.

We performed nested PCR to amplify five classes of Ig V_H_DJ_H_ transcripts and recombination activating gene-1 (RAG1) and RAG2 in isolated single PTECs. Peripheral blood mononuclear cells (PBMCs) were used as the positive control. Both RAG1 and RAG2 were detected in PTECs, suggesting similar mechanism of V(D)J rearrangement to that of B cells. Only the Igγ chain was detected in 82% of single PTECs (91/111), with 91.8% (45/49) in patient 1 and 74.2% (46/62) in patient 2. No Igα, μ, ε, or δ chain expression was detected (Fig. 2d,e and Fig. S1).

### PTEC-derived IgG presented some basic characteristics similar to those of B-IgG

To determine the characteristics of PTEC-derived IgG, 469 sequences of V_H_DJ_H_ rearrangements from 91 single PTECs were analyzed. The results showed that PTEC-derived IgG displayed a classic V_H_DJ_H_ rearrangement pattern with nucleotide additions at the V-D junctions and D-J junctions (Table 1). Among the 469 sequences examined, 27 (5.8%) were identified as nonfunctional because of mutations that introduced either stop codons into the variable region or out-of-frame V_H_DJ_H_ rearrangements (Table S2), which was similar to what was observed in B-IgG (3.9%, 130/3300).

**Table 1:** Ten sets of predominant V_H_DJ_H_ rearrangements with identical V–D junctions and D–J junctions were found in PTECs from different patients.

	V gene	V–D junctions	D gene	D–J junctions	J gene
1*	IGHV1-24	TT	IGHD2-15	ACCCGATCCGAC	IGHJ4
2*	IGHV1-18	TCTTAGTGGTTC	IGHD3-9		IGHJ6
3*	IGHV1-2	TCGGGGGG	IGHD3-3	CTTGGAGCCT	IGHJ6
4*	IGHV1-2	CTCTAT	IGHD3-9	AGCTGACA	IGHJ5
5*	IGHV1-3	CTTACC	IGHD3-22	GGATC	IGHJ4
6*	IGHV1-69	TCTGCCATTG	IGHD5-12	ATCCCCG	IGHJ4
7*	IGHV1-69	CCCTAAAG	IGHD2-15	GTTCTTTCTTGACGGCCC	IGHJ4
8*	IGHV1-8	CAACCGGGGCCAGCATACTG	IGHD3-3	CCTATGG	IGHJ5
9*	IGHV3-23	TAAATCAATCGATAT	IGHD3-3	GTTACGGA	IGHJ3
10*	IGHV4-59	TCGTTCA	IGHD3-22	CCGGAGTTTTTACCC	IGHJ3

1* was amplified in 1–3,1–6,1–15,1–19,1–21,1–29,1–39 and 2–10,2–11,2–18,2–30,2–42,2–45; 2* was amplified in 1–16,1–32,1–41 and 2–16; 3* was amplified in 1–8 and 2–37; 4* was amplified in 1–45 and 2–38; 5* was amplified in 1–17 and 2–43; 6* was amplified in 1–23 and 2–14; 7* was amplified in 1–20 and 2–44; 8* was amplified in 1–24 and 2–12; 9* was amplified in 1–22 and 2–26; and 10* was amplified in 1–43 and 2–2.

**Figure 3:**
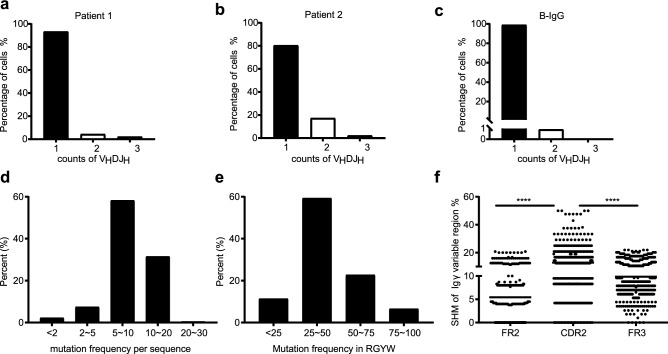
PTEC-derived IgG presents some basic characteristics similar to B-IgG. (**a**–**c**) Percentages of single cells expressing one, two, or three V_H_DJ_H_ segments. (**a**) In 45 single PTECs from patient 1 expressing IgG, two PTECs expressed two V_H_DJ_H_ patterns, and one expressed three V_H_DJ_H_ patterns. (**b**) In 46 single PTECs from patient 2 expressing IgG, eight PTECs expressed two V_H_DJ_H_ patterns, and one expressed three V_H_DJ_H_ patterns. (**c**) In 3084 single B cells expressing productive IgG in Shi’s article, 86 single B cells expressed two IgG V_H_DJ_H_ patterns, and none expressed three V_H_DJ_H_ patterns. (**d**–**f**) PTEC-derived IgG contained classic somatic hypermutation. Distribution of somatic hypermutations (**d**) and hotspot mutations (**e**) in Igγ variable region genes. Somatic hypermutations (**f**) were concentrated in the CDR but not in the FRs. A paired t-test was used to compare the mutation frequency between the CDR and FR. ****, p < 0.0001.

We analyzed the number of V_H_DJ_H_ rearrangement patterns in each single PTEC and found that 86.8% (79/91), 11.0% (10/91) and 2.2% (2/91) of single PTECs displayed one, two and more than two V_H_DJ_H_ rearrangement patterns, respectively, which was similar to what was observed in B-IgG, of which 97.2% displayed one V_H_DJ_H_ rearrangement and 2.8% showed two V_H_DJ_H_ rearrangement patterns (Fig. 3a–c).

In addition, we evaluated the mutational status of the Igγ variable genes in 469 V_H_DJ_H_ sequences from 91 single PTECs. Although we only amplified the Igγ variable gene segments from FR2 to JH regions, somatic hypermutations (SHM) were detected in the PTEC-derived IgG V_H_DJ_H_ gene in most sequences assessed (range from 2.9% to 20.5%), of which approximately 60% ranged from 5 to 10% (Fig. 3d). Given that antigen-induced SHM occurs mostly at the hotspot motif (RGYW/WRCY, W = A/T, R = A/G, Y = C/T), we analyzed hotspot mutations in PTEC-derived IgG and found that 90% of sequence mutations occurred in that hotpot motif (Fig. 3e). Moreover, similar to B cell-derived V_H_DJ_H_, somatic hypermutation was concentrated in the complementary determining region (CDR) and not in the framework region (FR) (Fig. 3f). These results indicate that PTEC-derived IgG usually undergoes classical somatic hypermutation.

### PTEC-derived IgG preferred VH1 in V_H_DJ_H_ rearrangements

**Figure 4:**
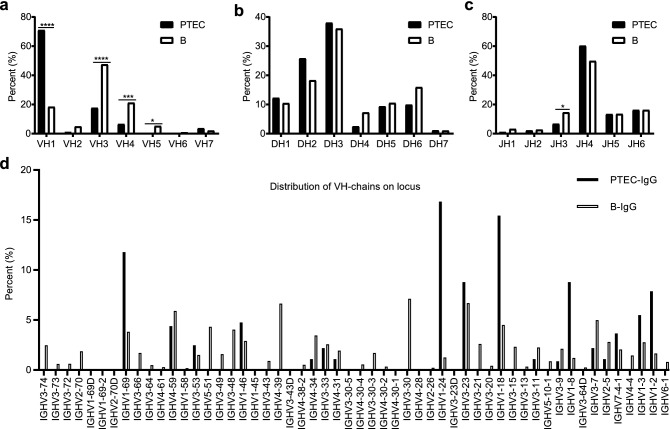
VH gene family usage in PTECs differs from that in B cells. (**a**) The VH gene family usage of PTECs differs significantly from that of B cells, especially higher percentage of VH1 in PTECs (****, p < 0.0001). The DH (**b**) and JH (**c**) gene family usage of PTECs are similar to those of B cells, except for lower percentage of JH3 in PTECs (*, p < 0.05). Chi-square test was used to compare the frequency of VH, D, JH usage between PTECs and B cells. (**d**) Distribution of the expressed VH genes of PTECs and B cells on the IgG heavy chain locus. Here, PTECs show a restricted distribution at the 3′ end compared to that of B cells, which express more widely distributed VH genes.

Analysis of VH gene usage indicated that 71% of the VH gene was VH1, while neither VH5 nor VH6 were amplified in PTECs; conversely, VH3 (47.5%) and VH4 (21.2%) were more frequently used in B cells (Fig. 4a). Of note, the three most commonly used VH genes in PTECs were IGHV1-24 (16.8%), IGHV1-18 (15.4%) and IGHV1-69 (11.8%), whereas B cells predominantly expressed IGHV3-30 (7.1%), IGHV3-23 (6.7%) and IGHV4-39 (6.6%) (Fig. S2a and S3a). Next, VH region genes were mapped regarding to their genome location for a comparative overview. The distribution of PTEC VH region genes on the genome was unilaterally concentrated at the 3′ end, whereas the VH genes in B cells displayed a uniform distribution (Fig. 4d).

All seven DH gene families and six JH gene families were amplified from PTECs, and the usage of segments and distribution of the gene families were overall similar to those of B cell-derived IgG with slight differences. (Fig. 4b,c, Fig. S2b-c, Fig. S3b-c and Fig. S4).

### PTEC-derived IgG displayed less diversity of V_H_DJ_H_ rearrangements than B-IgG

**Figure 5:**
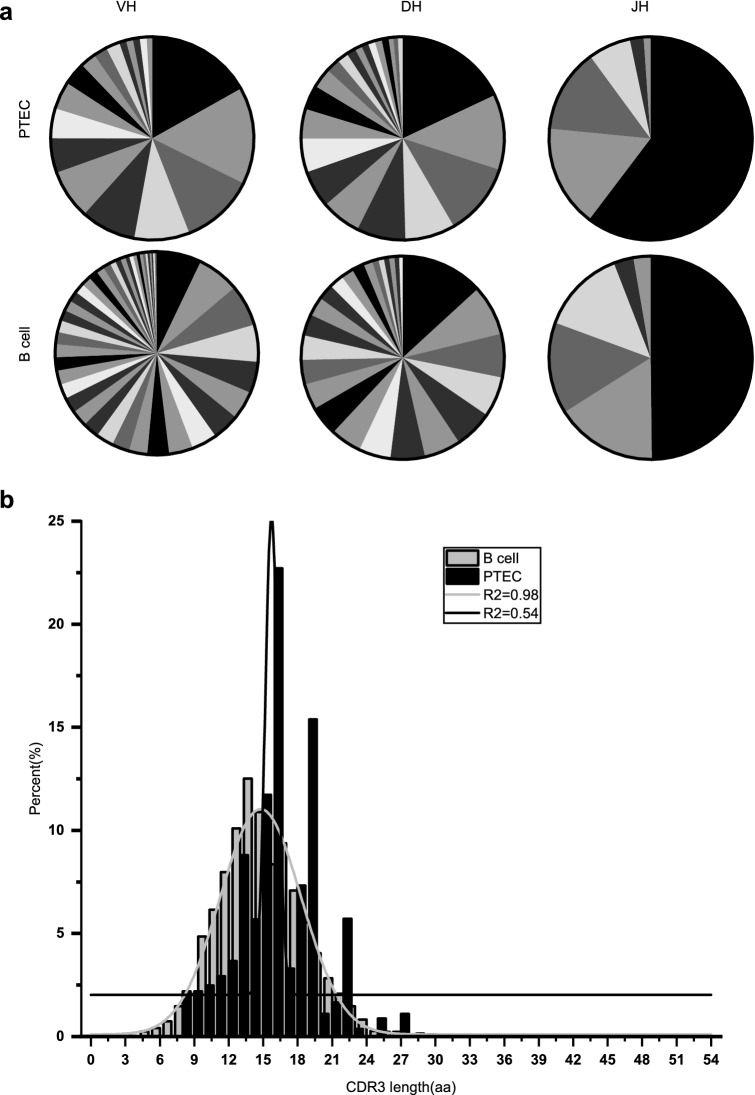
PTEC-derived IgG heavy chain repertoires are less diverse than those of B cells. (**a**) Igγ repertoire diversities in PTECs and B cells. Sector areas correspond to the relative frequency of each gene segment used in PTECs or B cells. More sectors represent more diversity. (**b**) The CDR3 length distribution of PTECs differs from that of B cells. The distribution of CDR3 length in normal B cells exhibit a Gaussian distribution (R^2^ = 0.98), while the distribution of CDR3 length in PTECs did not exhibit a Gaussian distribution (R^2^ = 0.54). *aa* amino acid.

**Figure 6:**
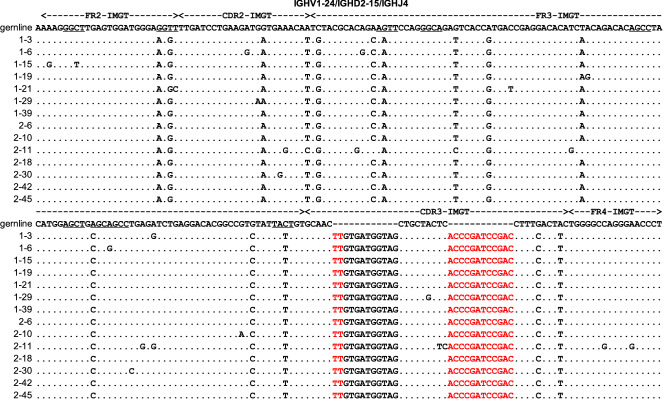
Predominant usage of VH1-24/DH2-15/JH4 rearrangements in different PTECs. The variable region sequences and mutations of VH1-24/DH2-15/JH4 are illustrated. These sequences of the V_H_DJ_H_ rearrangements were aligned and compared with the homologous germline sequences in a basic local alignment search tool (IgBLAST, https://www.ncbi.nlm.nih.gov/igblast/). Identity with the homologous germline sequence is indicated by dots. Each nucleotide mutation is indicated. The hotspot mutations in germline genes are underlined. The red letters refer to the junctions. CDR, complementarity determining region; FR, framework region.

Most single PTECs displayed their own unique V_H_DJ_H_ rearrangements, which were also relatively diverse in different individuals (Table S3 and S4). However, PTEC-derived IgG repertoires contained more conservative V_H_DJ_H_ rearrangements (Fig. 5a), with the VH1-24/DH2-15/JH4 rearrangement predominating in PTECs; this rearrangement pattern was found in 14.3% (7/49) of samples in patient 1 and 11.3% (7/62) in patient 2. Moreover, the same CDR3 sequences and similar SHMs were identified in the VH1-24/DH2-15/JH4 rearrangement from different individuals (Fig. 6). In addition, 10 pairs of single PTECs from different individuals and 5 pairs of single PTECs in the same individual shared the same V_H_DJ_H_ rearrangements with identical junctions (Table 1) (Table S5). All these results demonstrated limited diversity of PTEC-derived IgG repertoires.

In addition, the diversity of IgG can be obtained by measuring the length distribution of the CDR3 region. As shown in Fig. 5b, the length of CDR3 in normal B cells was close to a Gaussian distribution (R^2^ = 0.98), while the CDR3 length in PTECs was far from a Gaussian distribution (R^2^ = 0.54), suggesting that PTEC-derived IgG displayed less diversity than B-IgG.

## Discussion

In this study, we found for the first time that PTECs transcribe IgG and exhibit functional V_H_DJ_H_ rearrangements of IgG at the single-cell level. Similar to B-IgG, PTEC-derived IgG showed classic V_H_DJ_H_ rearrangements with nucleotide insertions at junctions and somatic hypermutation concentrated in the CDR. At the same time, PTEC-derived IgG demonstrates some distinct features, such as preferred VH1 usage, predominant V_H_DJ_H_ rearrangements in some single PTECs and skewed distribution of CDR3 lengths, suggesting that PTEC-derived IgG exhibits less diversity in V_H_DJ_H_ rearrangements than B-IgG.

Single-cell RNA sequencing (scRNA-seq) was developed to identify gene transcripts in a single cell. The Chromium system introduced by the 10 × Genomics Company has been widely used due to its high cell capture rate. In our study, only Igγ, κ and λ transcripts in both constant and variable regions were detected at a low level in a small number of PTECs, and none of the productive V(D)J rearrangements were amplified in the 10 × Genomics Chromium system, which may be explained by the fact that high-throughput scRNA-seq is not sensitive enough to detect low-abundance genes. Although it was difficult and slow to manually pick single PTECs sorted by FACS and to reconfirm their identity by limited marker gene expression, we successfully amplified the transcripts and V_H_DJ_H_ rearrangements of IgG in more than 80% of PTECs by combining nested PCR with Sanger sequencing. That no Igα, μ, ε, and δ chains were detected may be explained by the lack of Igα, μ, ε, δ chain transcripts or the low abundance of the transcripts in PTECs. In fact, we detected the transcripts of all five types of Ig heavy chains in glomerular podocytes and mesangial cells with V(D)J rearrangements (data not shown) by the same nested PCR with Sanger sequencing methodology.

Sequence analysis of PTEC-derived IgG V_H_DJ_H_ showed evidence of somatic hypermutations. The mechanism of SHM in PTEC-derived IgG V_H_DJ_H_ sequences may be similar to that in B cells. In B-Ig variable regions, SHM induced by antigen selection occurs more frequently in CDRs than in FRs, and AID site preference results in hypermutation at the RGYW hotspot motif. In this study, most of the V_H_DJ_H_ patterns matched the AID-induced RGYW hypermutation pattern. Moreover, SHM and hotspot mutations frequently occurred in the CDR but not in the FRs. Support of our results is that recombination activating gene 1 and 2 (RAG 1/2) and activation-induced cytidine deaminase (AID) transcripts were detected in many non-B cells, such as cancer cell lines^6^, as well as in a human podocyte cell line^22^. These results suggest that PTECs may express IgG in a similar mechanism to B cells.

In this study, some distinct features were found in PTEC-derived IgG. First, the VH genes in PTECs displayed biased VH1 usage (71% of the VH genes), with IGHV1-24, IGHV1-18 and IGHV1-69 as the most frequently used segments; this result was absolutely different from the usage of B cells in that VH3 and VH4 were the predominant usages, with IGHV3-30, IGHV3-23 and IGHV4-39 as the most frequently used segments. Second, PTEC-derived IgG had conservative V(D)J rearrangements, showing that VH1-24/DH2-15/JH4 was predominant in more than 10% of PTECs, 10 pairs of single PTECs from different individuals and 5 pairs of single PTECs from the same individual shared the same V_H_DJ_H_ rearrangements. All these are considered impossible in such a small number of B cells, and primer bias was excluded as the diversity of V(D)J rearrangements was proven in PBMCs (Table S6) or single B cells^24^. The phenomenon of conserved IgG characteristics has also been described in other non-B cells, such as human epithelial cancer cells^25^ or endothelial cells^26^. Our previous work, by Deng et al.^21^ and Jing et al.^22^, also found conservation in variable regions of IgA and IgG expressed in mesangial cells and podocytes, respectively.

Our work has some limitations. First, we only detected IgG transcripts in a small number of PTECs but failed to obtain enough information to analyze the V(D)J rearrangements by the 10 × Genomics Chromium system. Second, we did not detect light chain rearrangements in single PTECs, possibly due to low abundance of light chain genes. Third, we did not detect IgG protein in single PTECs. In our previous studies, IgA and IgG were detected by Western blot in both cell lysates and supernatants from human mesangial cells^21^ and podocytes^22^, which were reconfirmed by mass spectrometry. In fact, we detected the IgG protein expression and secretion in HK-2, an immortalized proximal tubular epithelial cell line (data not shown).

In conclusion, our results provide evidence for IgG transcripts and functional V_H_DJ_H_ rearrangements in single PTECs. PTEC-derived IgG presents not only basic characteristics similar to B-IgG but also exhibits less diversity and more conservation in V_H_DJ_H_ rearrangements than B-IgG. The potential role of PTEC‑derived IgG in the pathogenesis of nephropathy, especially in tubulointerstitial fibrosis, and its possible clinical implications require further investigation.

## Methods

### Patient samples and ethics statement

This study conformed to the principles of the Helsinki declaration. It was approved by the Medical Ethics Committee of Peking University Third Hospital (S2018207, Beijing, China) and was conducted in accordance with the protocol. Normal renal cortexes were collected from donors undergoing nephrectomy as a result of renal cell carcinoma. All donors voluntarily donated kidney cortexes and signed informed consent prior to donating the kidney cortex to the study. All methods were carried out in accordance with relevant guidelines and regulations. The inclusion criteria were as follows: age, 18–50 years old; blood pressure, 120–130/70–80 mmHg; proteinuria, negative; hematuria, non-glomerular original; serum creatinine, 62–115 μmol/L; and eGFR, 76–115 ml/min/1.73 cm^2^. Patients with a history of hepatitis B/C, diabetes, or rheumatic immune diseases before biopsy were excluded. A total of 3 samples were collected for the study, including one for the 10 × Genomics Chromium system and two for Sanger sequencing. The samples were strictly anonymized.

### 10 × library preparation and sequencing

Considering the abundance of tubular epithelial cells and the small number of glomerular intrinsic cells in the kidney cortex, the glomerulus was relatively enriched. A single cell suspension was then obtained from the normal kidney cortex. The concentration of the single-cell suspension was counted and adjusted to 1000 cells/μl for sorting 7000 cells. All remaining procedures, including library construction, were performed according to the manufacturer’s standard protocol described in Shi’s work^24^. We used the Cell Ranger software pipeline (version 3.0.0) to demultiplex cellular barcodes and map reads to the genome. Loupe Browser (version 4.0.0) was used for clustering. Loupe V(D)J Browser (version 3.0.0) was used for the V(D)J analysis of PTECs.

### Isolation of proximal tubular epithelial cells from the normal renal cortex

Human kidney samples of macroscopically normal cortical tissue were obtained from patients undergoing nephrectomy as a result of renal carcinoma. Renal cell isolation was performed within the subsequent 30 min. The renal cortex was decapsulated and minced with fine scissors into 1 mm^3^ fragments, and then it was digested with 1 mg/ml collagenase I in DMEM/F12 1:1 medium containing 1X ITS (insulin–transferrin–sodium selenite) at 37 °C for 20 min. The suspension was passed through filters with a mesh size of 70 μm after digestion. The cell suspension was washed twice in PBS and centrifuged for 5 min at 300 *g*.

Antibodies against two PTEC markers, CD10 and CD13, were used to isolate PTECs by FACS. 7AAD was used to eliminate dead cells. Renal cells were labeled with phycoerythrin (PE)-conjugated anti-CD13 and allophycocyanin (APC)-conjugated anti-CD10, incubated at 4 °C for 30 min and then washed twice in PBS. Cells were resuspended in 500 μl of PBS containing 0.04% BSA; then, they were sorted using BD FACS Aria II Special Order System and collected in PBS containing 0.04% BSA. Double positively labeled living cells were isolated and represented the PTEC population. The purity of the isolated cell fractions was confirmed by flow cytometry, the identity of the PTECs was validated by the specific marker gene LRP2, and the contamination of B cells was excluded by amplification of CD19 by PCR.

### Single PTEC dissociation and cDNA synthesis

Single PTECs were manually picked under a microscope by capillary pipette and then transferred to a 0.2 ml thin-wall PCR tube containing lysate buffer. Single PTEC RNA extraction and cDNA synthesis were carried out by Tang’s methods, as previously described^27^.

### PCR amplification

The variable regions of Ig heavy chain (Ig H) were amplified by nested PCR. Specifically, the complete V_H_DJ_H_ recombination of Ig H was amplified from single cell cDNA with an upstream variable-region primer pool for VH1-FR1, VH2-FR1, VH3-FR1, VH4-FR1, VH5-FR1 and VH6-FR1 and a downstream constant-region primer to amplify Ig H. For the second-round of PCR, an upstream primer that anneals to the framework 2 (FR2) region coupled with a JH primer was used to amplify the variable region of Ig H. Touchdown PCR was performed for single cell Ig H amplification. Nested PCR was also performed to detect the PTEC-specific marker gene LRP2 and the B cell marker gene CD19. The primer pool of the upstream variable-region refers to primers in Biomed-2^28^. Other primers used for PCR are listed in Table S7.

### Cloning and Sanger sequencing

For the generation of the Igγ sequences, PCR products were cloned into a pGEM-T Easy Vector (Promega, Madison, WI) and then were transformed into DH5a-competent bacteria (Invitrogen). In total, 4–8 colonies per sample were randomly chosen and sequenced with an ABI 3100 Genetic Analyzer (Applied Biosystems, Foster City, CA). The rearranged V_H_DJ_H_ sequences were compared with those in a basic local alignment search tool (IgBLAST, https://www.ncbi.nlm.nih.gov/igblast/) to identify the best matching germline gene segments and junctions following primer trimming. For the analysis of somatic hypermutation, part of the IgH V region from FR2 to JH was used. The mutation status was defined as mutated if there were 2% or more mutations compared with the germline sequences.

### Data mining of the B cell IgG repertoire

To compare the characteristics of the immune repertoire between PTECs and B cells at the single-cell level, we obtained V_H_DJ_H_ sequence information of B cell-derived IgG in a previous article by Shi^24^. We screened 3187 IgG^+^ B cells and obtained 3300 V_H_DJ_H_ sequences (including 130 unproductive sequences). The frequency and distribution of VH, D, and JH usage in single B cells was analyzed and compared with that of single PTECs.

### Statistical analysis

Statistical analysis was performed using GraphPad Prism 7.0. A paired t-test was used to compare the mutation frequency between the CDR and FR. Chi-square test was used to compare the frequency of VH, D, JH usage between PTECs and B cells *p < 0.05 and ****p < 0.0001 were considered statistically significant.

## Supplementary information

Supplementary Information

## Data Availability

For original data, please contact bjwangyue@sina.com.
