# Supplementary material for: Single-cell RNA sequencing confirms IgG transcription and limited diversity of VHDJH rearrangements in proximal tubular epithelial cells
Source: Sci Rep. 2020 Nov 12;10:19657. doi: 10.1038/s41598-020-75013-9 (PMC7661700; doi:10.1038/s41598-020-75013-9)

**Single cell RNA sequencing confirms IgG transcription and limited diversity of V_H_DJ_H_ rearrangements in proximal tubular epithelial cells**

Zhenling Deng^1^, Xinyao Wang^1^, Yue Liu^1^, Xinyu Tian^1^, Shaohui Deng^1^, Yingchun Sun^1^, Song Wang^1^, Danxia Zheng^1^, Zhuan Cui^1^, Yuejuan Pan^1^, Lata A^1^, Huige Yan^2^, Xiaoyan Qiu^2*^, Yue Wang^1*^

1. Department of Nephrology, Peking University Third Hospital, Beijing 100191, China.

2. Department of Immunology, School of Basic Medical Sciences, Peking University, Beijing, 100191, China

Correspondence to:

Professor Yue Wang, Department of Nephrology, Peking University Third Hospital, 49 Huayuanbei Road, Beijing 100191, P.R. China

E-mail: bjwangyue@sina.com

Professor Xiaoyan Qiu, Department of Immunology, School of Basic Medical Sciences, Peking University, 38 Xueyuan Road, Beijing 100191, P.R. China

E-mail: [qiuxy@bjmu.edu.cn](mailto:qiuxy@bjmu.edu.cn)

Running headline: Human PTECs express IgG

Table S1. Clinicopathologic features of 2 patients with renal carcinoma assessed for IgG transcripts and V_H_DJ_H_ rearrangements.

| Case | Age/Sex | Blood pressure (mmHg) | proteinuria | Serum creatinine (μmol/L) | ABL  (g/L) | eGFR (ml/min/1.73m^2^) |
| --- | --- | --- | --- | --- | --- | --- |
| Patient 1 | 31/M | 118/71 | negative | 100 | 41.2 | 87 |
| Patient 2 | 46/F | 113/79 | negative | 68 | 39.4 | 93 |

Table S2. Rate of non-functional V_H_DJ_H_ rearrangements.

| Case | No. of PTECs | No. of IgG^+^ PTEC (%) | No. of functional V_H_DJ_H_ rearrangements | No. of non-functional V_H_DJ_H_ rearrangements (%) |
| --- | --- | --- | --- | --- |
| Patient 1 | 49 | 45(91.8%) | 236 | 9 (3.7%) |
| Patient 2 | 62 | 46(74.2%) | 206 | 18(8.0%) |
| Total | 111 | 91 (82%) | 442 | 27(5.8%) |

Table S3. The V_H_DJ_H_ rearrangement patterns of IgG heavy chain variable region in 45 single PTECs from patient 1.

| Cell | Top V gene | Top D gene | Top J gene | score | clone | productive |
| --- | --- | --- | --- | --- | --- | --- |
| 1-1 | IGHV1-46 | IGHD5-12 | IGHJ4 | 86.5-87.1 | 6/6 | Yes |
| 1-2 | IGHV1-18 | IGHD6-19 | IGHJ4 | 91.5-92.7 | 6/6 | Yes |
| 1-3 | IGHV1-24 | IGHD2-15 | IGHJ4 | 91.7 | 6/6 | Yes |
| 1-4 | IGHV1-2 | IGHD1-1 | IGHJ5 | 89.5-90.6 | 6/6 | Yes |
| 1-5 | IGHV1-8 | IGHD2-21 | IGHJ4 | 84.7-85.9 | 6/6 | Yes |
| 1-6 | IGHV1-24 | IGHD2-15 | IGHJ4 | 89.9-90.5 | 6/6 | Yes |
| 1-7 | IGHV3-7 | IGHD1-1 | IGHJ5 | 90.6 | 5/5 | Yes |
| 1-8 | IGHV1-2 | IGHD3-3 | IGHJ6 | 90.5 | 5/5 | Yes |
| 1-9 | IGHV4-59 | IGHD2-15 | IGHJ2 | 86.1 | 5/5 | Yes |
| 1-10 | IGHV3-7 | IGHD6-19 | IGHJ4 | 92.4-94.2 | 5/5 | Yes |
| 1-11 | IGHV3-23 | IGHD6-19 | IGHJ6 | 100 | 5/5 | Yes |
| 1-12 | IGHV4-59 | IGHD3-22 | IGHJ3 | 91.1-91.7 | 6/6 | Yes |
| 1-13 | IGHV1-2 | IGHD2-2 | IGHJ4 | 83-83.6 | 6/6 | Yes |
| 1-14 | IGHV1-18 | IGHD3-3 | IGHJ6 | 94.1-94.7 | 6/6 | Yes |
| 1-15 | IGHV1-24 | IGHD2-15 | IGHJ4 | 90.5-91.7 | 5/5 | Yes |
| 1-16 | IGHV1-18 | IGHD3-9 | IGHJ6 | 93.5-94.7 | 6/6 | Yes |
| 1-17 | IGHV1-3 | IGHD3-22 | IGHJ4 | 89.3-90.1 | 5/5 | Yes |
| 1-18 | IGHV3-33 | IGHD1-1 | IGHJ6 | 93.6 | 6/6 | Yes |
| 1-19 | IGHV1-24 | IGHD2-15 | IGHJ4 | 91.1-91.7 | 6/6 | Yes |
| 1-20 | IGHV1-69 | IGHD2-15 | IGHJ4 | 87.7-88.3 | 2/2 | Yes |
| 1-21 | IGHV1-24 | IGHD2-15 | IGHJ4 | 91.1-91.7 | 5/5 | Yes |
| 1-22 | IGHV3-23 | IGHD3-3 | IGHJ3 | 86.6 | 1/1 | Yes |
| 1-23 | IGHV1-69 | IGHD5-12 | IGHJ4 | 92.3-92.9 | 5/5 | Yes |
| 1-24 | IGHV1-8 | IGHD3-3 | IGHJ5 | 95.3 | 2/2 | Yes |
| 1-25 | IGHV1-46 | IGHD5-12 | IGHJ4 | 88.3-88.9 | 6/6 | Yes |
| 1-26 | IGHV1-8 | IGHD2-2 | IGHJ2 | 93.5-94.7 | 5/5 | Yes |
| 1-27 | IGHV1-18 | IGHD3-3 | IGHJ4 | 84.1-85.4 | 6/6 | Yes |
| 1-28 | IGHV1-18 | IGHD3-3 | IGHJ6 | 93.9-94.7 | 6/6 | Yes |
| 1-29 | IGHV1-24 | IGHD2-15 | IGHJ4 | 91.7-92.3 | 5/5 | Yes |
| 1-30 | IGHV2-5 | IGHD1-26 | IGHJ5 | 95.7-95.8 | 6/6 | Yes |
| 1-31 | IGHV1-69 | IGHD4-17 | IGHJ5 | 88.8-95.5 | 3/6 | Yes |
| 1-31 | IGHV1-3 | IGHD5-12 | IGHJ3 | 97.1 | 1/6 | Yes |
| 1-31 | IGHV1-69 | IGHD3-10 | IGHJ6 | 95.3 | 2/6 | Yes |
| 1-32 | IGHV1-18 | IGHD3-9 | IGHJ6 | 94.1-94.7 | 6/6 | Yes |
| 1-33 | IGHV7-4-1 | IGHD2-8 | IGHJ4 | 90.2-90.9 | 2/2 | Yes |
| 1-34 | IGHV1-18 | IGHD3-16 | IGHJ5 | 83.2-83.8 | 4/4 | Yes |
| 1-35 | IGHV1-8 | IGHD6-19 | IGHJ4 | 94.1-95.3 | 4/4 | Yes |
| 1-36 | IGHV3-23 | IGHD3-3 | IGHJ1 | 80.7-81.3 | 6/6 | Yes |
| 1-37 | IGHV1-2 | IGHD7-27 | IGHJ4 | 88.9 | 5/5 | Yes |
| 1-38 | IGHV3-11 | IGHD6-25 | IGHJ4 | 82.8-83.4 | 4/4 | Yes |
| 1-39 | IGHV1-24 | IGHD2-15 | IGHJ4 | 91.1-92.3 | 6/6 | Yes |
| 1-40 | IGHV1-18 | IGHD1-26 | IGHJ4 | 92.2 | 2/6 | Yes |
| 1-40 | IGHV1-46 | IGHD5-24 | IGHJ4 | 78.9-80.1 | 4/6 | Yes |
| 1-41 | IGHV1-18 | IGHD3-9 | IGHJ6 | 94.1 | 6/6 | Yes |
| 1-42 | IGHV1-18 | IGHD3-3 | IGHJ6 | 92.9-94.1 | 4/5 | Yes |
| 1-42 | IGHV1-69 | IGHD6-19 | IGHJ4 | 92.9 | 1/5 | Yes |
| 1-43 | IGHV4-59 | IGHD3-22 | IGHJ3 | 91.6-92.3 | 6/6 | Yes |
| 1-44 | IGHV7-4-1 | IGHD1-26 | IGHJ4 | 87.1-88.1 | 4/4 | Yes |
| 1-45 | IGHV1-2 | IGHD3-9 | IGHJ5 | 93.5-94.7 | 6/6 | Yes |

1-1 refers to the first cell of Patient 1, 1-2 refers to the second cell of Patient 1, and so on.

Table S4. The V_H_DJ_H_ rearrangement patterns of IgG heavy chain variable region in 46 single PTECs from Patient 2.

| Cell | Top V gene | Top D gene | Top J gene | score | clone | productive |
| --- | --- | --- | --- | --- | --- | --- |
| 2-1 | IGHV1-18 | IGHD1-1 | IGHJ6 | 95.3 | 1/2 | Yes |
| 2-1 | IGHV1-3 | IGHD3-10 | IGHJ4 | 90.5 | 1/2 | Yes |
| 2-2 | IGHV4-59 | IGHD3-22 | IGHJ3 | 88.7-90.5 | 3/3 | Yes |
| 2-3 | IGHV1-69 | IGHD5-12 | IGHJ4 | 89.9-90.5 | 5/5 | Yes |
| 2-4 | IGHV1-69 | IGHD3-9 | IGHJ4 | 92.4 | 4/4 | Yes |
| 2-5 | IGHV1-8 | IGHD5-12 | IGHJ4 | 89-91.2 | 4/4 | Yes |
| 2-6 | IGHV1-24 | IGHD2-15 | IGHJ4 | 92.3 | 6/6 | Yes |
| 2-7 | IGHV3-23 | IGHD1-26 | IGHJ4 | 91.5-91.6 | 5/5 | Yes |
| 2-8 | IGHV4-31 | IGHD3-22 | IGHJ3 | 88.1-89.9 | 4/4 | Yes |
| 2-9 | IGHV3-53 | N/A | IGHJ4 | 92.8 | 5/5 | Yes |
| 2-10 | IGHV1-24 | IGHD2-15 | IGHJ4 | 91.7 | 5/5 | Yes |
| 2-11 | IGHV1-24 | IGHD2-15 | IGHJ4 | 89.9-90.5 | 5/5 | Yes |
| 2-12 | IGHV1-8 | IGHD3-3 | IGHJ5 | 93.6-94.7 | 6/6 | Yes |
| 2-13 | IGHV1-69 | IGHD3-22 | IGHJ6 | 90.6-91.2 | 5/5 | Yes |
| 2-14 | IGHV1-69 | IGHD5-12 | IGHJ4 | 91.2-92.3 | 6/6 | Yes |
| 2-15 | IGHV7-4-1 | IGHD1-26 | IGHJ4 | 94.7-95.3 | 6/6 | Yes |
| 2-16 | IGHV1-18 | IGHD3-9 | IGHJ6 | 94.1 | 6/6 | Yes |
| 2-17 | IGHV1-24 | IGHD3-22 | IGHJ4 | 93.3 | 5/6 | Yes |
| 2-17 | IGHV1-2 | IGHD6-25 | IGHJ4 | 99.1 | 1/6 | Yes |
| 2-18 | IGHV1-24 | IGHD2-15 | IGHJ4 | 92.3 | 4/4 | Yes |
| 2-19 | IGHV1-8 | IGHD1-14 | IGHJ4 | 86.5-87.1 | 3/3 | Yes |
| 2-20 | IGHV3-23 | IGHD1-26 | IGHJ4 | 83.8-85 | 5/5 | Yes |
| 2-21 | IGHV3-53 | IGHD2-21 | IGHJ4 | 81.4 | 1/4 | Yes |
| 2-21 | IGHV1-18 | IGHD4-17 | IGHJ4 | 94.1-94.7 | 3/4 | Yes |
| 2-22 | IGHV3-53 | IGHD2-21 | IGHJ4 | 79.6-80.8 | 5/6 | Yes |
| 2-22 | IGHV3-53 | IGHD6-25 | IGHJ4 | 80.2 | 1/6 | Yes |
| 2-23 | IGHV1-24 | IGHD3-22 | IGHJ4 | 92.1-93.3 | 2/4 | Yes |
| 2-23 | IGHV1-69 | IGHD6-19 | IGHJ4 | 87-87.6 | 2/4 | Yes |
| 2-24 | IGHV1-69 | IGHD2-21 | IGHJ5 | 83-83.6 | 6/6 | Yes |
| 2-25 | IGHV4-34 | IGHD3-10 | IGHJ4 | 89.9 | 1/1 | Yes |
| 2-26 | IGHV3-23 | IGHD3-3 | IGHJ3 | 85.9-86 | 3/3 | Yes |
| 2-27 | IGHV1-46 | IGHD6-6 | IGHJ4 | 90.6-91.2 | 6/6 | Yes |
| 2-28 | IGHV1-3 | IGHD3-22 | IGHJ5 | 87.6-88.2 | 2/6 | Yes |
| 2-28 | IGHV1-18 | IGHD5-18 | IGHJ4 | 98.2 | 4/6 | Yes |
| 2-29 | IGHV1-3 | IGHD5-12 | IGHJ5 | 91.2 | 1/1 | Yes |
| 2-30 | IGHV1-24 | IGHD2-15 | IGHJ4 | 90.5-91.7 | 5/5 | Yes |
| 2-31 | IGHV1-18 | IGHD3-10 | IGHJ4 | 95.3-95.9 | 2/2 | Yes |
| 2-32 | IGHV1-69 | IGHD3-22 | IGHJ4 | 92.3-92.9 | 4/4 | Yes |
| 2-33 | IGHV3-33 | IGHD6-13 | IGHJ4 | 84.2-86 | 5/5 | Yes |
| 2-34 | IGHV1-69 | IGHD2-21 | IGHJ4 | 79.5-80.7 | 6/6 | Yes |
| 2-35 | IGHV1-46 | IGHD3-16 | IGHJ5 | 81.7-81.9 | 4/6 | Yes |
| 2-35 | IGHV1-18 | IGHD2-21 | IGHJ5 | 93.5 | 1/6 | Yes |
| 2-35 | IGHV1-18 | IGHD3-16 | IGHJ5 | 89.3 | 1/6 | Yes |
| 2-36 | IGHV1-18 | IGHD4-11 | IGHJ6 | 84.5-85.1 | 4/6 | Yes |
| 2-36 | IGHV7-4-1 | IGHD1-1 | IGHJ4 | 88.3 | 2/6 | Yes |
| 2-37 | IGHV1-2 | IGHD3-3 | IGHJ6 | 90.5-91.1 | 3/3 | Yes |
| 2-38 | IGHV1-2 | IGHD3-9 | IGHJ5 | 93.5-94.1 | 6/6 | Yes |
| 2-39 | IGHV1-3 | IGHD3-10 | IGHJ4 | 89.3-90 | 3/3 | Yes |
| 2-40 | IGHV3-23 | IGHD1-1 | IGHJ4 | 85.9 | 4/4 | Yes |
| 2-41 | IGHV1-8 | IGHD6-19 | IGHJ4 | 96.5-97.1 | 5/5 | Yes |
| 2-42 | IGHV1-24 | IGHD2-15 | IGHJ4 | 92.3 | 2/2 | Yes |
| 2-43 | IGHV1-3 | IGHD3-22 | IGHJ4 | 90.6-91.2 | 5/5 | Yes |
| 2-44 | IGHV3-9 | IGHD3-22 | IGHJ6 | 97.1-97.6 | 4/5 | Yes |
| 2-44 | IGHV1-69 | IGHD2-15 | IGHJ4 | 87.4 | 1/5 | Yes |
| 2-45 | IGHV1-24 | IGHD2-15 | IGHJ4 | 91.7-92.3 | 5/5 | Yes |
| 2-46 | IGHV3-23 | IGHD3-16 | IGHJ6 | 85.8-86.5 | 3/3 | Yes |

2-1 refers to the first cell of Patient 2, 2-2 refers to the second cell of Patient 2, and so on.

Table S5. Five pairs of single PTECs shared the same V_H_DJ_H_ in an individual.

|  | V gene | V-D junctions | D gene | D-J junctions | J gene |
| --- | --- | --- | --- | --- | --- |
| 1* | IGHV1-3 | GAAAGG | IGHD3-10 | CTAATGGG | IGHJ4 |
| 2* | IGHV3-53 |  | IGHD2-21 | GGCGGCG | IGHJ4 |
| 3* | IGHV1-46 | GAGGGGGAGAA | IGHD5-12 | CCCT | IGHJ4 |
| 4* | IGHV4-59 | TCGTTCA | IGHD3-22 | CCGGAGTTTTTACCC | IGHJ3 |
| 5* | IGHV1-18 | TCTTACAGGTTCACGATCCCTT | IGHD3-3 |  | IGHJ6 |

1* was amplified in 2-1 and 2-39; 2* was amplified in 2-21 and 2-22; 3* was amplified in1-1 and 1-25; 4* was amplified in1-12 and1-43; 5* was amplified in1-14 and1-28.

Table S6. The V_H_DJ_H_ rearrangement patterns of Ig heavy chains variable region in PBMCs.

| Ig class | Top V gene | Top D gene | Top J gene | score | clone | productive |
| --- | --- | --- | --- | --- | --- | --- |
| Ig γ | IGHV3-53 | IGHD2-15 | IGHJ4 | 91.7 | 1/8 | Yes |
|  | IGHV3-23 | IGHD1-1 | IGHJ4 | 86.7 | 1/8 | Yes |
|  | IGHV3-7 | IGHD6-13 | IGHJ4 | 94.1 | 2/8 | Yes |
|  | IGHV3-15 | IGHD3-3 | IGHJ6 | 92.4 | 1/8 | Yes |
|  | IGHV3-21 | IGHD1-1 | IGHJ4 | 89.1 | 1/8 | Yes |
|  | IGHV3-48 | IGHD2-21 | IGHJ6 | 95.3 | 1/8 | Yes |
|  | IGHV3-53 | IGHD7-27 | IGHJ6 | 84.2 | 1/8 | Yes |

Table S7. Primers used in this study.

| Gene | Primer | Primer sequence 5′-3′ |
| --- | --- | --- |
| IgG heavy chain variable region | Sense primer pool (VH1-VH6) | refer to primers in Biomed-2^[28]^ |
|  | Antisense primer (IGHGc-R1) | GAGTCCTGAGGACTGTAGGACAG |
|  | Internal sense primer (VH-FR2) | TGGRTCCGVCAGSCYCCNGG |
|  | Internal antisense primer (JH) | AACTGCAGAGGAGACGGTGACC |
| PTEC specific marker genes | External sense primer (LRP2) | GGAGGTGCAGACCTAAAGGAG |
|  | External antisense primer (LRP2) | CACAACAGCGCAGCCAAT |
|  | Internal sense primer (LRP2) | GTGCAGACCTAAAGGAGCGTT |
|  | Internal antisense primer (LRP2) | ATGCAATGCCCACTTCCACA |
|  | External sense primer (CD10) | GCCTCTGCCTTGGGGAGTTAT |
|  | External antisense primer (CD10) | ACGTTTCAACCAGCCTCCG |
|  | Internal sense primer (CD10) | GGGAATTTGCCATTCTGCTGTA |
|  | Internal antisense primer (CD10) | AGCCTCCGCAAGCATATTTGA |
|  | External sense primer (CD13) | CCCAGCTCCACACACCGTT |
|  | External antisense primer (CD13) | ATTGGGGGTGAGGTACGGTC |
|  | Internal sense primer (CD13) | CTCCACACACCGTTCCTGGAT |
|  | Internal antisense primer (CD13) | GCGATGATTGTGCACACGG |
| B cell marker gene | External sense primer (CD19) | GAGGGAGATAGGCACGGATGG |
|  | External antisense primer (CD19) | AACATTGCTCCAGAGGTTGGC |
|  | Internal sense primer (CD19) | GTGGCTACTGGCTTTCAGG |
|  | Internal antisense primer (CD19) | CAGGTGTGAATCTTGGGGACTT |
| RAG1 | External sense primer (RAG1) | TGGATCTTTACCTGAAGATG |
|  | External antisense primer (RAG1) | CTTGGCTTTCCAGAGAGTCC |
|  | Internal sense primer (RAG1) | CACAGCGTTTTGCTGAGCTC |
|  | Internal antisense primer (RAG1) | AGCTTGCCTCAGGGTTCATG |
| RAG2 | External sense primer (RAG2) | TGGAAGCAACATGGGAAATG |
|  | External antisense primer (RAG2) | CATCATCTTCATTATAGGTGTC |
|  | Internal sense primer (RAG2) | TTCTTGGCATACCAGGAGAC |
|  | Internal antisense primer (RAG2) | CTATTTGCTTCTGCACTG |


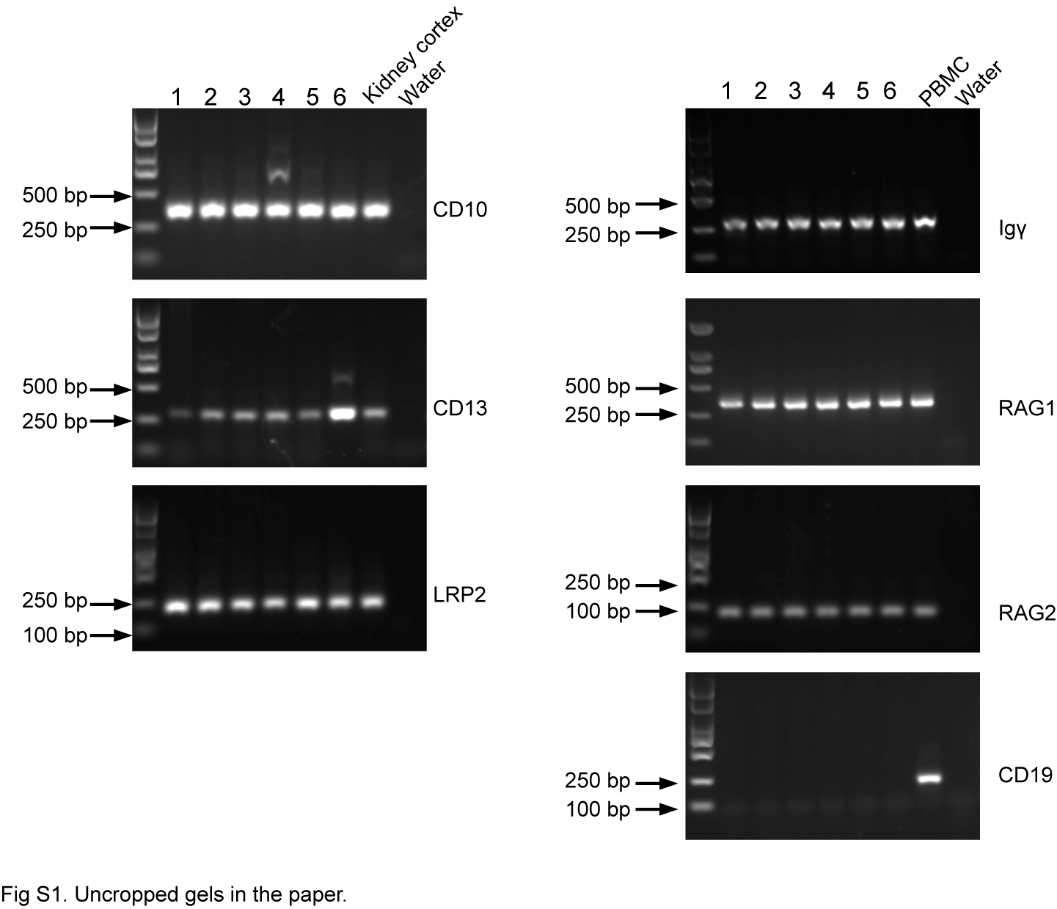


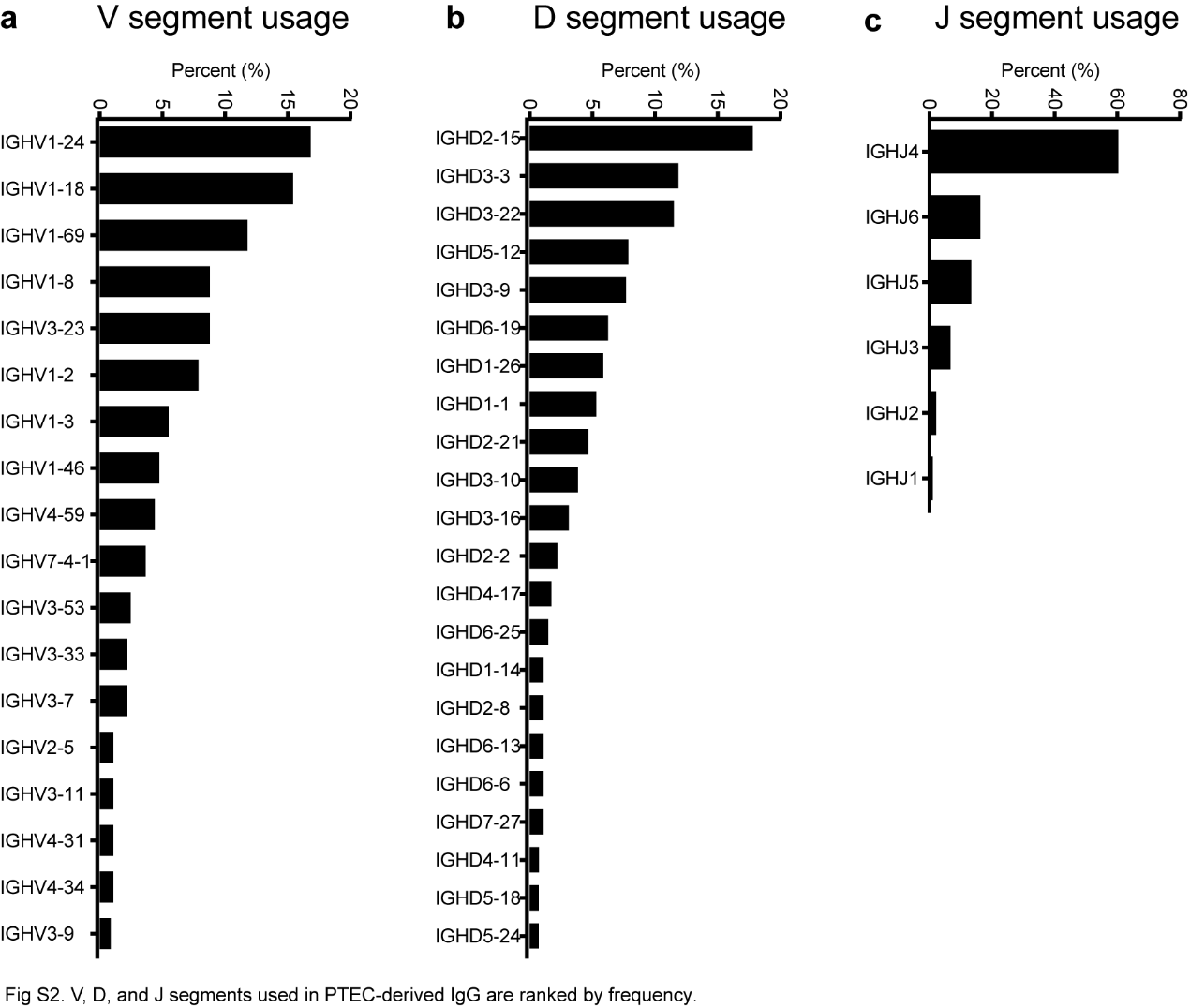


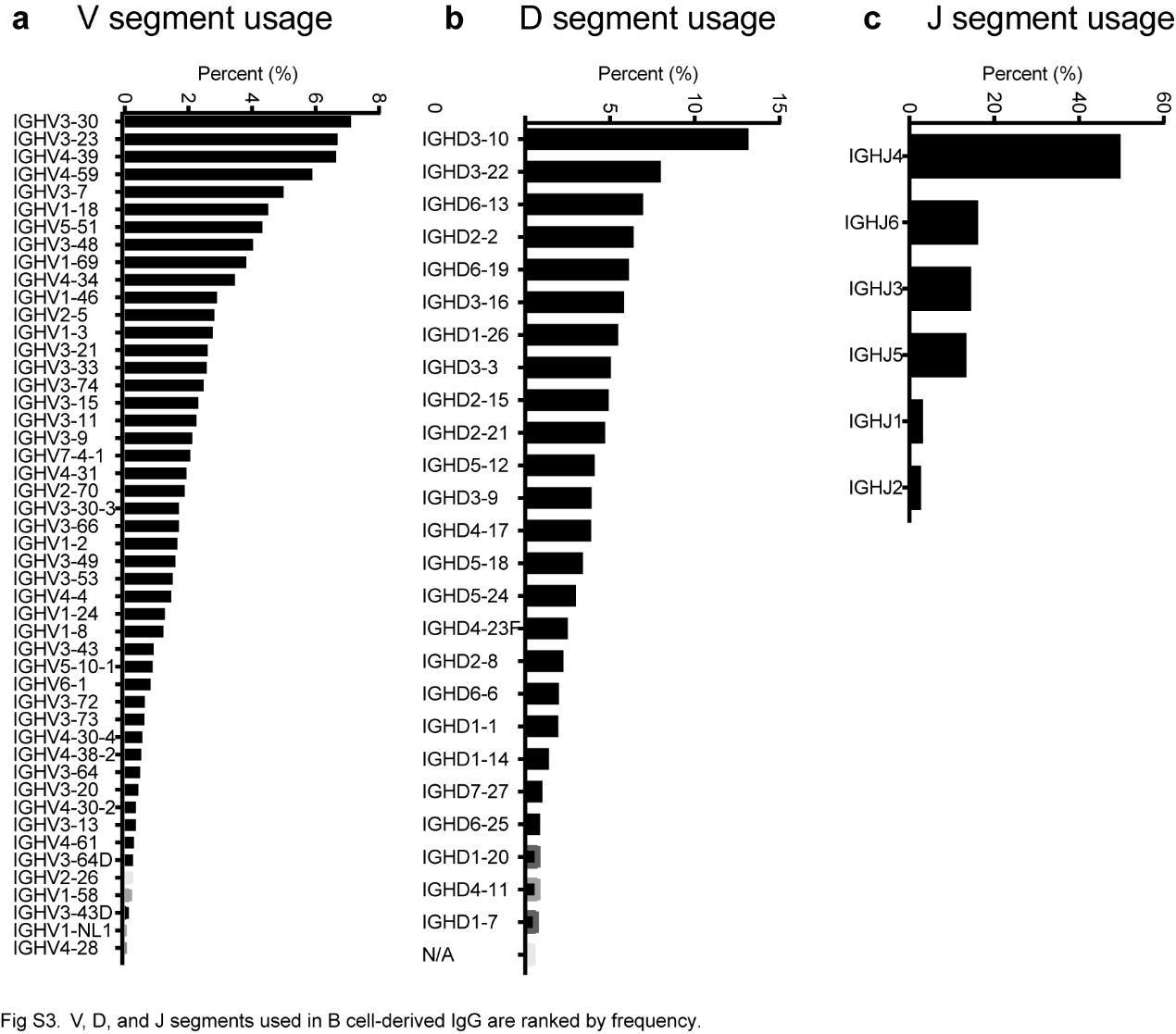

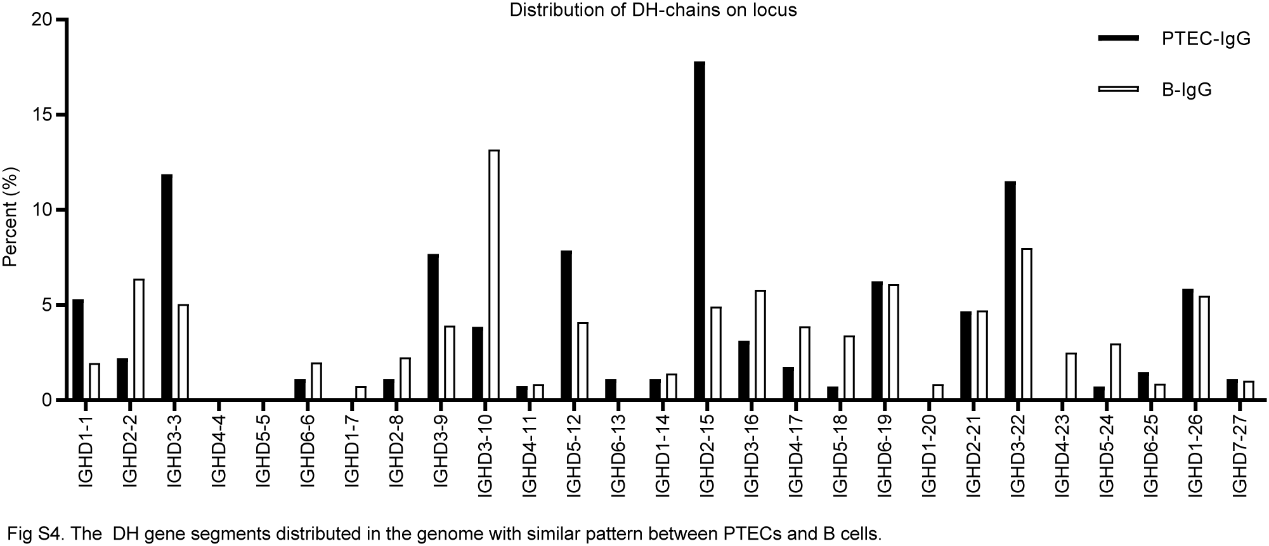

Supplement: Supplementary file 1 — Supplementary Information [file 41598_2020_75013_MOESM1_ESM.docx]
